# Characterization of Carbonic Anhydrase 9 in the Alimentary Canal of *Aedes aegypti* and Its Relationship to Homologous Mosquito Carbonic Anhydrases

**DOI:** 10.3390/ijerph14020213

**Published:** 2017-02-21

**Authors:** Daniel P. Dixon, Leslie Van Ekeris, Paul J. Linser

**Affiliations:** 1 The Whitney Laboratory, University of Florida, Saint Augustine, FL 32080, USA; leslie@whitney.ufl.edu (L.V.E.); pjl@whitney.ufl.edu (P.J.L.); 2 The Anastasia Mosquito Control District, St. Augustine Florida, Saint Augustine, FL 32092, USA

**Keywords:** carbonic anhydrase (CA), mosquito, alkaline, pH, midgut, immunohistochemistry, data mining, molecular phylogenetics

## Abstract

In the mosquito midgut, luminal pH regulation and cellular ion transport processes are important for the digestion of food and maintenance of cellular homeostasis. pH regulation in the mosquito gut is affected by the vectorial movement of the principal ions including bicarbonate/carbonate and protons. As in all metazoans, mosquitoes employ the product of aerobic metabolism carbon dioxide in its bicarbonate/carbonate form as one of the major buffers of cellular and extracellular pH. The conversion of metabolic carbon dioxide to bicarbonate/carbonate is accomplished by a family of enzymes encoded by the carbonic anhydrase gene family. This study characterizes *Aedes aegypti* carbonic anhydrases using bioinformatic, molecular, and immunohistochemical methods. Our analyses show that there are fourteen *Aedes aegypti* carbonic anhydrase genes, two of which are expressed as splice variants. The carbonic anhydrases were classified as either integral membrane, peripheral membrane, mitochondrial, secreted, or soluble cytoplasmic proteins. Using polymerase chain reaction and Western blotting, one of the carbonic anhydrases, *Aedes aegypti* carbonic anhydrase 9, was analyzed and found in each life stage, male/female pupae, male/female adults, and in the female posterior midgut. Next, carbonic anhydrase 9 was analyzed in larvae and adults using confocal microscopy and was detected in the midgut regions. According to our analyses, carbonic anhydrase 9 is a soluble cytoplasmic enzyme found in the alimentary canal of larvae and adults and is expressed throughout the life cycle of the mosquito. Based on previous physiological analyses of adults and larvae, it appears AeCA9 is one of the major carbonic anhydrases involved in producing bicarbonate/carbonate which is involved in pH regulation and ion transport processes in the alimentary canal. Detailed understanding of the molecular bases of ion homeostasis in mosquitoes will provide targets for novel mosquito control strategies into the new millennium.

## 1. Introduction

In the mosquito midgut, luminal pH regulation is important for the digestion of food. Digestive proteases are secreted into the midgut lumen to process detritus in mosquito larvae and the blood meals in female adults. These proteases are highly active at neutral and alkaline pH [1]. The midgut lumen of mosquito larvae and adults is alkaline [2,3], and the major buffer molecule in the midgut lumen of mosquitoes is bicarbonate (HCO_3_^−^). Luminal HCO_3_^−^ and carbonate (CO_3_^2−^) levels are regulated by a network of proteins in mosquitoes which may include carbonic anhydrase (CA), a zinc metaloenzyme that hydrates carbon dioxide (CO_2_) to form HCO_3_^−^ and protons (H^+^) [4,5]. In this report, we characterized the fourteen isoforms of *Aedes aegypti* CA via bioinformatic gene sequence analysis, data mining of transcriptomes [6], and molecular phylogenetics. We also analyzed *Aedes aegypti* CA9 (AeCA9), one of the highly expressed cytoplasmic CAs, using molecular biology and immunohistochemical analyses. More specifically, AeCA9 mRNA expression and protein localization were analyzed in the different life stages along with adult digestive and ion regulatory tissues.

There are six different evolutionary gene families of CA: alpha, beta, gamma, delta, zeta, and eta [7,8] Also, there are five different types of CAs, each with different spatial localizations: soluble cytoplasmic, soluble secreted, integral membrane, mitochondrial, and glycosylphosphatidyl inositol (GPI)—anchored [5]. CA isozymes also have different activity levels due to differences in amino acid sequence, active site structure, and post-translational modifications [9,10,11]. Despite catalyzing the same chemical reaction, different isoforms are associated with a vast array of physiological processes [12,13,14]. Various solute carriers and ion transporters such as members of the SLC4 protein family and energizing transporters such as H+ V-ATPase and metabolic enzymes such as the CAs are hypothesized to influence gut luminal pH and maintain ion homeostasis in larval and adult mosquito guts [3,4,15,16,17,18,19].

**Figure 1:**
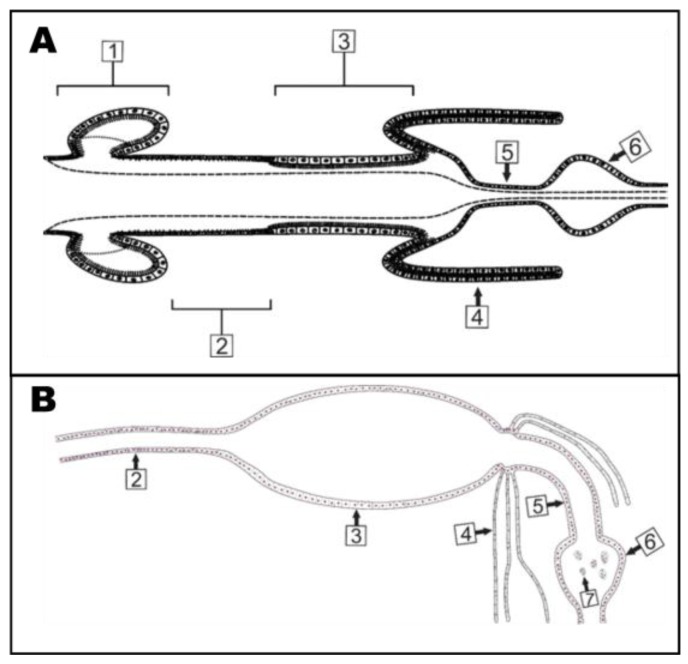
Anatomy of the larval and adult mosquito gut. (**A**) The larval alimentary canal is comprised of the foregut and cardia (not shown), GC (1), AMG (2), PMG (3), MTs (4), ileum (5), and rectum (6) (adapted from [5]); (**B**) The adult alimentary canal is comprised of the AMG (2), PMG (3), MTs (4), ileum (5), rectum (6), and rectal papillae (7).

In the larvae, the midgut is described as having three regions: the gastric caeca (GC), anterior midgut (AMG), and posterior midgut (PMG) [20] (Figure 1A). The GC are eight lobed extensions of the midgut epithelium which occupy much of the internal space of the thorax of the larvae and are the gut epithelial extensions just posterior to the cardia. Caecal epithelia are made up of different cell types: absorptive/secretory cells, ion transport cells, regenerative cells, cap cells, and neurosecretory cells [19,21,22,23,24]. Luminal pH within the caecal lobes is ~8 [2]. The AMG is mainly composed of large epithelial cells along with relatively few regenerative and neurosecretory cells. In the AMG lumen of *Aedes aegypti* larvae, the pH reaches 10.5–11 as a relatively focal region within the gut-long gradient of pH [2]. The epithelial cells of the PMG, like most cells of the GC, have extensive apical microvilli which likely play roles in the absorption of nutrients and secretion of cellular products (i.e., enzymes) [2,25]. The luminal pH of the PMG is closer to neutrality at ~7.6 to 8.5 [2]. Physiological analyses of CA function in the larval gut pH are conflicted. CA activity was detected in the GC and PMG in the larvae of *Aedes aegypti* using ^18^O exchange and Hansson′s histochemical stain [26]. When whole mosquito larvae are fed methazolamide (a CA inhibitor), the pH gradient inside the gut is compromised [16,26]. Yet, when isolated and perfused AMG and PMG tissues are exposed to CA inhibitors, there is no change in AMG alkalinity or PMG acid transport [18,27]. Thus, it appears that although CA activity certainly produces some of the ionic milieu that participates in the luminal pH gradient, the driving force for the pH extremes is provided by transporters, most likely H+V-ATPase, which expend ATP in the vectorial energization of the process [18,23,28].

In adult mosquitoes, the midgut is divided into two main regions: the AMG and PMG (Figure 1B). In females, the AMG is a site of sugar digestion [29] while the PMG is a site for both sugar and blood meal digestion [30]. Also, the PMG is the initial site of ion regulation between the midgut lumen and hemolymph following a blood meal [31]. In males, the AMG also digests sugar but the exact function of the PMG is unknown [20]. The adult female mosquito maintains the PMG lumen at a pH of 8.5–9.5 [3], but the pH in the male alimentary canal remains unknown. To test if the products of CA activity play a role in gut pH regulation in female adults, ^18^O exchange and Hansson′s Histochemical stain were used and detected CA activity in the AMG and PMG [3]. CA activity was also inhibited in the female PMG using 100 µM methazolamide which decreased the pH in the PMG lumen [3]. Finally, in isolated PMG tissues CA activity was inhibited and this resulted in a decrease in fluid reabsorption [17].

There is a major gap in our knowledge of CA expression and characterization in *Aedes aegypti*. Only three isoforms of mosquito CA have been characterized: *Anopheles gambiae* CA10 (AgCA10) [24], *Aedes aegypti* CA10 (AeCA10) (ibid), and AgCA9 [4]. The remaining CA isoforms in both species are uncharacterized. CAs have been analyzed in terms of their capacity to influence the pH in the adult gut lumen [3] and effect proton transport following a blood meal [32]. In both of those studies, the isoforms of CA in the midgut that could be involved with ion transport and pH regulation were not determined. Recent research discusses the expression and physiology of HCO_3_^−^ transporters [19,33], yet the producers of HCO_3_^−^, CA, have not been well characterized, especially in the major arbovirus vector *Aedes aegypti*. It should also be noted that the role of CA is simple: reversibly convert metabolic/catabolic CO_2_ into H_2_CO_3_ which readily ionizes into forms that can be moved vectorially by ion transport systems. CO_2_ as a non-polar gas, can diffuse through membranes and create uncontrolled havoc in compartmentalized biological systems. Successful aerobic respiration requires the controlled movement of this ultimate by product of catabolism. Thus, localization of CA activity has implications on much more than simply pH regulation.

This study characterizes *Aedes aegypti* CAs (AeCA), with emphasis on the gut, to determine which CAs are expressed in the different life stages and gut regions of the mosquito. This information will enhance our understanding of the overall regulation of metabolic CO_2_. We report that carbonic anhydrases are differentially expressed in the life stages and across both sexes in *Aedes aegypti*. First, RNA transcript sequences and predicted protein sequences of every AeCA isoform were analyzed in silico to reveal sequence motifs that inform about CA physiology, i.e., subcellular compartmentalization and membrane attachment. Next, the expression of each AeCA isoform was analyzed via a transcriptomics [6] to determine which CAs are most highly abundant in each life stage and tissue. AeCA9, one of the most highly expressed CAs in the Akbari et al., 2013 transcriptome [6] and orthologous to AgCA9 [4], was further analyzed via polymerase chain reaction (PCR) in each sex, life stage (larvae, pupae, and adults), and within the PMG and MTs of adult females. Protein expression of AeCA9 in the same life stages and tissues was evaluated using Immunoblotting. Finally, AeCA9 protein was examined using immunohistochemistry in the larvae along with male and female adults, specifically in digestive and ion regulatory tissues. We report that AeCA9 is compartmentalized to the epithelial cells of the alimentary canal of larvae and adults. We also integrate this information into models which include previously published analyses of relevant membrane transporters to the pH regulatory paradigm.

## 2. Materials and Methods

### 2.1. Mosquito Rearing

*Aedes aegypti* were reared to adulthood in 1:1 tap/deionized H_2_O (dH_2_O) under a 12 h day-light cycle. Mosquito larvae were fed a yeast-liver powder solution (10 g yeast, 15 g liver powder, 500 mL water) every other day. Adult mosquitoes were fed 10% sucrose in dH_2_O. Mosquito larvae were preserved for tissue sections at the early 4th instar stage and adult mosquitoes were dissected for SDS PAGE and Western blotting and preserved for tissue sectioning 3–10 days post eclosion. Before all dissections, mosquitoes were cold-anesthetized at 4 °C for a minimum of 10 min and rested on ice.

### 2.2. Data Mining and Bioinformatics

AeCA genes were previously identified in a phylogenetic analysis from [4] and any new CAs were identified based on orthology to *Anopheles* gambiae using the database Vectorbase [34]. Al AeCA transcript and protein sequences were acquired from the Vectorbase gene expression browser [34]. Analyses of CA protein sequences for structural and functional domains (transmembrane domain, signal peptides, etc.) were conducted using Vectorbase, Interproscan [35] and Phobius [36] WoLF PSORT was used to predict subcellular localization of each predicted CA protein sequence [37].

CA transcript expression was quantified from the transcriptome developed by Akbari et al. 2013 [6], and RPKM expression data was mined from the Akbari et al. 2013 supplementary tables. Next, the RPKM expression levels were analyzed on TIBCO Spotfire software (Boston, MA, USA, 2015). A heat map of CA expression was generated using a pre-determined color scheme that changes in intensity relative to the level of CA expression. TIBCO analyses were exported onto the Corel Draw X6 (Ottawa, ON, Canada) graphics suite for editing.

The molecular phylogeny of AeCA protein sequences was conducted using MEGA 6 [38]. The protein sequences were aligned using MUSCLE [39], and a Maximum Likelihood Tree was generated using an LG model where the rates among sites were gamma distributed with invariant sites (G + I). We used the bootstrap method to test the phylogeny with 100 bootstrap replications.

### 2.3. Polymerase Chain Reaction (PCR)

In this section, unless otherwise stated, the number of biological replicates for each life stage and tissue group was three, and the number of animals per biological replicate was ten for each life stage and 15 for each tissue group. An exception was the pupae (male and female) with five animals analyzed per biological replicate. RNA was extracted from each of the life stage/tissue samples using TRI-reagent (Molecular Research Center, Cincinnati, OH, USA) and purified using Directzol RNA columns (Zymogen, Irvine, CA, USA). The quantity and purity of each RNA sample was analyzed using a nanodrop spectrophotometer, and the quality of the RNA was analyzed using a non-denaturing 1% Tris/Borate/EDTA (TBE) (Thermo Scientific, Waltham, MA, USA) agarose gel with ethidium bromide (ETBR). For each of the above samples, cDNA was synthesized from up to 1 µg of purified total RNA using Superscript III reverse transcriptase (Life Technologies, Carlsbad, CA, USA). 1 ng of cDNA was used in each PCR, and the samples were run for 30 cycles using the protocol from Dreamtaq Polymerase (Thermo Scientific). Primers were designed via Primer 3.1 targeting AeCA9. Forward and Reverse primers were designed using Integrated DNA Technologies (Coralville, IA, USA): Forward primer –ATGTCGACCTCTTGGGGATAC, Reverse primer –TCAGTGTCCTCCGAATTCGC, melt temperature was 60 degrees. The PMG and MT samples were run for 36 cycles. Each sample was further analyzed on a 1% agarose gel with ETBR in TBE buffer and visualized using a GeneFlash Bioimager (Syngene, Frederick, MD, USA).

### 2.4. Western Blots

While RNA was extracted from the samples listed above, protein was simultaneously extracted from each life stage/sex group following the manufacturer′s instructions for TRI-reagent (Molecular Research Center Inc., Cincinnati, OH, USA) unless otherwise stated. Each life stage/tissue group was evaluated using three biological replicates, and each biological replicate is comprised of 5–15 mosquitoes (for MTs, there are 5 MTs per mosquito). The protein content for each sample was quantified using a Pierce BCA protein assay kit according to the manufacturer′s instructions (Thermo Scientific). For each life stage/sex replicate, 6 μg of total protein was loaded into each lane. For the posterior midgut (PMG) and Malpighian tubules (MT) samples, 40 μg of protein was loaded in each lane. For the tissue groups (PMG, MT), protein was extracted using a urea-based homogenization buffer (8 M urea (Sigma, St. Louis, MO, USA), 2 M thiourea (Sigma), 4% (w/v) CHAPS (Sigma) and 30 mM Tris (Sigma) adjusted to pH 8.5) and subsequently precipitated using acetone. Following acetone precipitation, the proteins were solubilized in the same buffer as the TRI-reagent isolated proteins above. The solubilized proteins were also supplemented with 1% protease inhibitor cocktail (Sigma). Isolated protein was run on a 4%–12% NuPAGE Bis-tris polyacrylamide gel (Life Technologies, Carlsbad, CA, USA) according to manufacturer′s instructions, then transferred to a nitrocellulose membrane. The non-affinity purified primary chicken IgY targeting AeCA9 [4] was diluted 1000X in blotto (5% dry milk, 0.15% Triton X-100 in Tris-buffered saline (TBS)). After the blot was blocked for 1 h at room temperature in blotto, the blot was incubated with the primary antibody overnight in blotto at 4 °C. The blot was washed in TBS, then incubated in blotto with Alkaline-phosphatase conjugated donkey anti-chicken secondary antibodies (Jackson Immunoresearch, West Grove, PA, USA), diluted 1000X. Finally, the blot was developed using an alkaline phosphatase development kit (Biorad, Hercules, CA, USA).

### 2.5. Immunohistochemical Analyses

Mosquitoes were prepared for paraffin embedding according to Smith et al. 2008 [40]. Briefly, primary fixation of mosquitoes was done by direct injection into the hemocoel with 4% paraformaldehyde in PBS with subsequent immersion in 4% paraformaldehyde in PBS overnight at 4 °C. Next, preserved animals were immersed in Carnoy′s solution (60% ethanol, 30% chloroform, 10% glacial acetic acid) for 90 min on ice then washed twice with 100% ethanol for 30 min each. The mosquitoes were then immersed in a 1:1 mixture of aniline: methyl salicylate overnight followed by 100% methyl salicylate overnight. Mosquitoes were then embedded in paraffin wax and stored at room temperature until ready to use. The sections cut were 6–10 microns thick and mounted onto charged slides. Sections were rehydrated by standard methods [40]. The slides were then incubated in pre-incubation medium (0.3% BSA, 1% Normal goat serum, 0.15% triton-X 100 in TBS) for 1 h at room temperature, then overnight with antibodies diluted in pre-incubation medium at 4 °C. The following polyclonal and monoclonal antibody dilutions were used: AgCA9 IgY at 1:100–1:500 and the monoclonal antibody to the alpha subunit of Sodium-Potassium ATPase (NaK-ATPase) (Developmental Studies Hybridoma bank, [41]) at 1:10. The NaK-ATPase antibody is a mouse monoclonal IgG generated against the whole alpha five subunit of the chicken NaK-ATPase (Developmental studies Hybridoma Bank). NaK-ATPase was used for this study as a reliable marker of basal membranes in gut tissues and some ion regulatory tissues. The slides were then washed with TBS and incubated with the following secondary antibodies (Jackson Immunoresearch, West Grove, PA, USA) diluted 1:250 in pre-incubation medium: FITC-conjugated donkey anti-chicken IgY, TRITC-conjugated goat anti-mouse IgG. The slides were incubated with the secondary antibodies for 2 h at 37 °C. After washing with TBS, cover slips were applied to the slides using 60% glycerol in TBS supplemented with p-phenylenediamine (PPD) to reduce signal quenching. Slides were analyzed on a Leica SP5 confocal microscope. Images were assembled into figures using Corel Draw X6 graphics suite.

### 2.6. Preabsorption Controls

The antibodies to AeCA9 were pre-absorbed with the peptide used to manufacture the antibody. The diluted IgY was pre-absorbed in pre-incubation medium without Triton X-100 (0.3% BSA, 1% Normal goat serum in TBS). The synthesized peptide (Thermo Scientific) was dissolved to 1 mg/mL then diluted to 100 µg/mL in the triton-free pre-incubation medium. A control for the absorption assay was water instead of peptide. AeCA9 IgY was diluted 250× for both the control and pre-absorption controls and incubated overnight at 4 °C. Standard protocols from above were followed for Western blots and Immunohistochemical analyses using the absorbed and control antibody solutions. For Western blots, the pre-absorbed/control antibodies were used at a final dilution of 1:1000. For Immunohistochemical analyses on tissue sections, the final dilution was 1:500.

## 3. Results

### 3.1. Predicted Transcript and Protein Sequence Information for Aedes aegypti CAs

In this report, the genomic, transcript, and protein sequences of all 14 *Aedes aegypti* CAs were analyzed and compared [34,35,36,38,42]. Many of the *Aedes aegypti* CAs that were analyzed are orthologous to *Anopheles gambiae* CAs. All of the data concerning sequence length (DNA, RNA, and protein), signal peptides, transmembrane helices, and orthology is presented in Table 1 and Table 2. Vectorbase identifiers for AeCAs (i.e., AAEL004930) along with the identifiers from Smith et al. [4] are used to name the CAs based on orthology to *Anopheles gambiae*. Also, a new molecular phylogeny against the CA genes from six species (four mosquitoes, *Drosophila*, and Human) was generated (discussed later), yielding similar results to the Smith et al. 2007 phylogeny [4]. Finally, all WoLF PSORT predictions for subcellular compartmentalization were included in Table 2.

#### 3.1.1. AeCA Genome and Transcript Sequence Analysis

In this study, the sizes of each AeCA gene were documented on Table 1. Only 8 of the 14 CA sequences were mapped on to the three chromosomes due to the misassembly of *Aedes* CA sequences [42]. Two CA genes were mapped to Chromosome 1, three CA genes were mapped to chromosome 2, and three CA genes were mapped to chromosome 3. The three CA genes on chromosome 2 (*AeCA-RP5*, *AeCA9*, and *AeCA4*) were grouped in approximately the same locus. On chromosome 3, two of the CA genes (*AAEL000843* and *AeCAβ*) were also grouped to approximately the same locus.

The predicted transcripts of CA ranged in length from 822 bp to 2609 bp, and two of the CA genes are expressed as splice variants: *AeCA3* and *AeCA7*. In *AeCA3*, the splice variation is found within the 5′ UTR. In *AeCA3-RA* the 5′ UTR is 147 bp in length, and in *AeCA3-RB* the 5′ UTR is 632 bp in length. The 5′ UTR of both splice variants is identical within the last 83 bp, but the sequences upstream of the last 83 bp are divergent (Supplementary Materials Figure S1). The putative protein sequence translated by both *AeCA3* splice variants is the same. In *AeCA7*, the splice variation is also found within the 5′ UTR. In *AeCA7-RA*, the 5′ UTR is 253 bp in length while in *AeCA7-RB* the 5′ UTR is 695 bp in length. The sequence of the 5′-UTRs in both splice variants of *AeCA7* have low similarity (Supplementary Materials Figure S2). The *AeCA7* splice variants each code for the same putative protein.

#### 3.1.2. AeCA Protein Sequence Analysis

The predicted isoforms of CA differ in amino acid number; they range in length from 216 AA to 483 AA (Table 1). Thirteen of the fourteen *Aedes aegypti* CA genes code for alpha CAs, while one codes for a beta CA (*AeCAβ*). Predicted protein sequences were analyzed to determine the number of isoforms that are classified as soluble cytoplasmic, soluble secreted, integral membrane, mitochondrial, and peripheral membrane (Table 2). The two protein domains searched for in each sequence were transmembrane helices (to indicate membrane localization) and signal peptides. Soluble cytoplasmic CAs lack both transmembrane helices and signal peptides. Five of the CA proteins are predicted to be soluble cytoplasmic according to Phobius, Interproscan, and WoLF PSORT (AeCAβ, AAEL000843, AeCA8, AeCA9, and AeCA-RP2). Soluble secreted CAs lack a transmembrane domain but have a signal peptide. Four of the predicted CA proteins have been classified as secreted CAs based on the presence of a signal peptide (AeCA6, AAEL010886, AeCA7, and AAEL010894). There are three CAs that have transmembrane domains: AeCA4, AeCA10, and AAEL009330. Two of these isoforms, AeCA10 [24] and AAEL009330, have both a signal peptide and a transmembrane domain, indicating they are secreted to a cellular compartment or the cell membrane. One of those isoforms, AeCA10, was previously characterized as a GPI-linked peripheral membrane protein [24]. Analyses using big-PI predictor [43] indicated that AAEL009330 is also a GPI-anchored CA. AeCA4 was predicted to have just a transmembrane domain according to Phobius. Two CA proteins, AeCA-RP5 and AeCA3, are predicted mitochondrial CAs according to WoLF PSORT. AeCA-RP5 is a putative carbonic anhydrase related protein (CA-RP), orthologous to the *Anopheles gambiae* AgCA-RP5.

### 3.2. CA Transcript Expression across Development and Sex of Aedes aegypti

**Figure 2:**
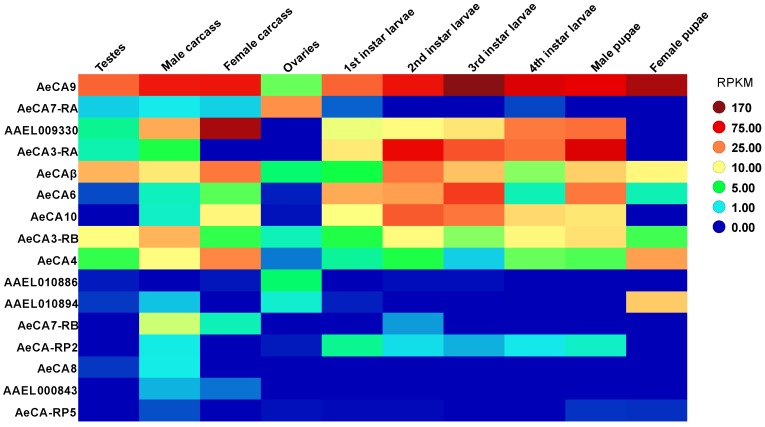
Transcriptomic analysis of CA in each life stage via RNA-seq [6]. The rows indicate each CA gene, while each column is a life stage analyzed from the [6] transcriptome. The color on the heat map represents the relative expression in Reads per Kilobase per Million Mapped Reads (RPKM) of each CA in each tissue. The more hot colors (reds and orange) represent more abundant expression, while cooler colors (blues and greens) represent less abundant expression. A legend to the right of the heat map provides a reference for the color code to RPKM levels.

**Figure 3:**
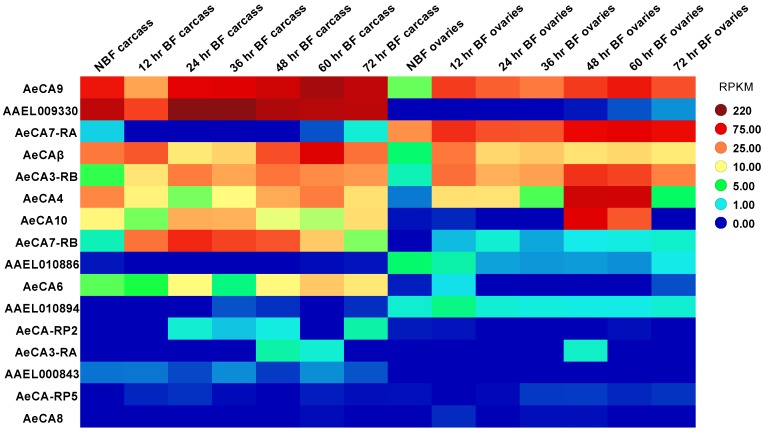
Transcriptomic analysis of CA in the carcass and ovaries post blood meal via RNA-seq [6]. The rows indicate each CA gene, while each column is a blood meal time point in either the carcass or ovaries analyzed from the [6] transcriptome. The color on the heat map represents the relative expression in Reads per Kilobase per Million Mapped Reads (RPKM) of each CA in each tissue and time point. The more hot colors (reds and orange) represent more abundant expression, while cooler colors (blues and greens) represent less abundant expression. A legend to the right of the heat map provides a reference for the color code to RPKM levels.

Akbari et al. 2013 [6] published a transcriptome in which all the life stages of *Aedes aegypti* were analyzed along with sex-specific organs and blood fed tissues. For this paper, we will only look at the expression data for the 4th instar larvae, male and female pupae, male carcass and testes, and female carcass and ovaries (carcass is defined as all tissues except the sex organs) [6]. The 14 isoforms of CA have variable expression levels in the different life stages/organs, but not all CAs are abundant according to the heat map. We determined that the following CA transcripts were expressed abundantly for each life stage (Figure 2): *AeCA9*, *AeCA7-RA*, *AAEL009330*, *AeCA3-RA*, *AeCAβ*, *AeCA6*, *AeCA10*, *AeCA3-RB*, and *AeCA4*. Similarly, the blood meal time series for the carcass and ovaries [6] was also analyzed. We determined the following CA transcripts were expressed abundantly in the carcass and ovaries, though their expression was not always similar between both tissues (Figure 3): *AeCA9*, *AAEL009330*, *AeCA7-RA*, *AeCAβ*, *AeCA3-RB*, *AeCA4*, *AeCA10*, *AeCA7-RB*, and *AeCA6*. The transcript with the greatest expression level in most tissues analyzed was *AeCA9*, and this isoform will be the primary focus of the remainder of this manuscript.

### 3.3. A Molecular Phylogeny of AeCA Protein Sequences

**Figure 4:**
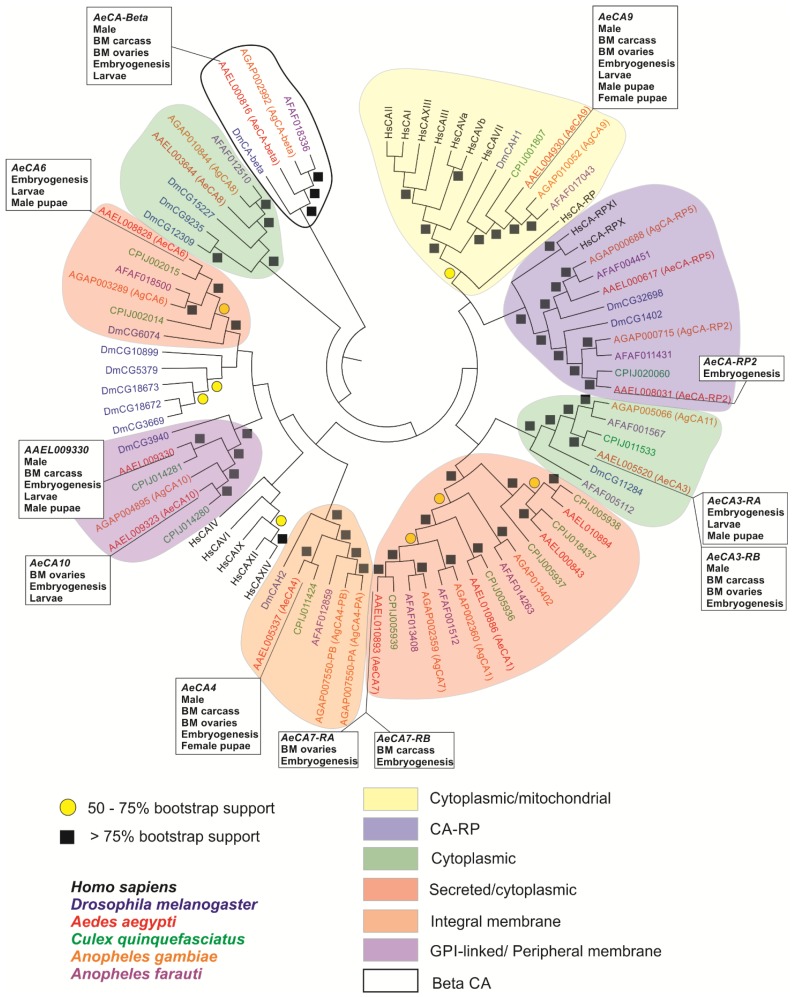
Molecular phylogeny of *Aedes aegypti* CA isoforms. This maximum likelihood molecular phylogeny was generated using MEGA 6 and based on a MUSCLE alignment of *Homo sapiens*, *Drosophila melanogaster*, *Aedes* *aegypti*, *Culex* *quinquefasciatus*, *Anopheles* *gambiae*, and *Anopheles farauti*. Bootstrap values are represented by yellow circles (50%–75% bootstrap) and black squares (75%–100% bootstrap). Colored bubbles encircle branches of the phylogeny where CAs with similar cellular compartmentalization or structure were grouped together. A legend below the phylogeny shows which color pertains to which compartmentalization/structure. This phylogeny correlates well with the [4] phylogeny and now shows the relationship of *Aedes aegypti* CAs to *Culex quinquefasciatus* and *Anopheles farauti* CAs together with the other species. Finally, the tissue and cellular compartments within which each AeCA was expressed [6] is marked on the phylogeny to correlate RNA expression with the molecular phylogeny. The species color code is as follows: *Homo sapiens* (black), *Drosophila melanogaster* (blue), *Aedes aegypti* (red), *Culex quinquefasciatus* (green), *Anopheles gambiae* (orange), and *Anopheles farauti* (purple).

**Figure 5:**
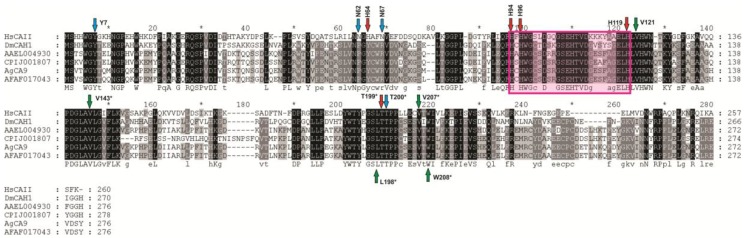
MUSCLE alignment of AeCA9 and its orthologues. AeCA9 (AAEL004930) was aligned with its orthologous sequences in *Homo sapiens* (HsCAII), *Drosophila melanogaster* (DmCAH1), *Culex quinquefasciatus* (CPIJ001807), *Anopheles gambiae* (AgCA9), and *Anopheles farauti* (AFAF017043). *Homo sapiens* CAII was used as a guide to map out the residues in *Aedes aegypti* that make up the active site and support active site structure (red arrows). Also, amino acids that stabilize the active site water molecules (blue arrows) and form the CO_2_ binding pocket (green arrows) were mapped onto the alignment. The residues are highlighted in a graded fashion using the following shades that represent amino acid percent identity: Black (100% identity), Dark gray (80% identity), Light gray (60% identity), no shade (no similarity). The pink box overlaying the alignment indicates where the Human CAII active site is relative to the insect CAs (Human His94 through His119).

A maximum likelihood molecular phylogeny was generated against each CA protein sequence from six species: *Homo sapiens*, *Drosophila melanogaster*, *Anopheles gambiae*, *Anopheles* *farauti*, *Culex* *quinquefasciatus*, and *Aedes* *aegypti* (Figure 4). There were distinct sets of CAs that grouped based on cellular compartmentalization and are visualized on the tree. Finally, this new molecular phylogeny agrees with the previously published mosquito CA phylogeny [4] as AeCA9, the mosquito CA of focus in this manuscript, aligns with both *Drosophila* and Human CAs indicating it is the CA that most closely resembles the ancestral CA between protostomes and deuterostomes. The protein sequences of each CA that grouped with AeCA9 were aligned on a MUSCLE alignment (Figure 5), and each amino acid involved in the formation of the active site, water chain stabilization, and the CO_2_ binding pocket were mapped onto the alignment. All of these amino acids could be found lined up with the human CA, though there were some insect specific modifications to the amino acids involved in the formation of the water chain and the proton shuttle [44]. In human CAs, the proton shuttle is a histidine at position 64, but according to the alignment in insect cytoplasmic CAs the proton shuttle is a tyrosine at position 66. This histidine to tyrosine shift is found in all the insects analyzed in the alignment (*Drosophila*, *Aedes* *aegypti*, *Culex* *quinquefasciatus*, *Anopheles* *gambiae*, and *Anopheles* *farauti*).

### 3.4. AeCA9 Transcript and Protein Expression across the Development and Sex of Aedes aegypti

**Figure 6:**
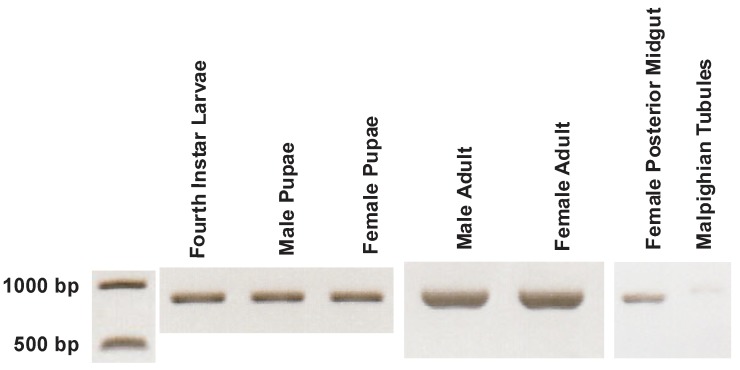
PCR of AeCA9 in each life stage, female posterior midgut, and female Malpighian tubules. The transcript for AeCA9 is 831 bp, and the 1 KB DNA ladder (left) indicates the size of the PCR product. The PCR for each life stage and the posterior midgut was run for 30 cycles and is non-quantitative, while the PCR for the Malpighian tubules were run for 36 cycles and was also not quantitative. AeCA9 was detected in all the life stages analyzed, and AeCA9 transcript was expressed in the PMG and is barely detectable in the MTs.

**Figure 7:**
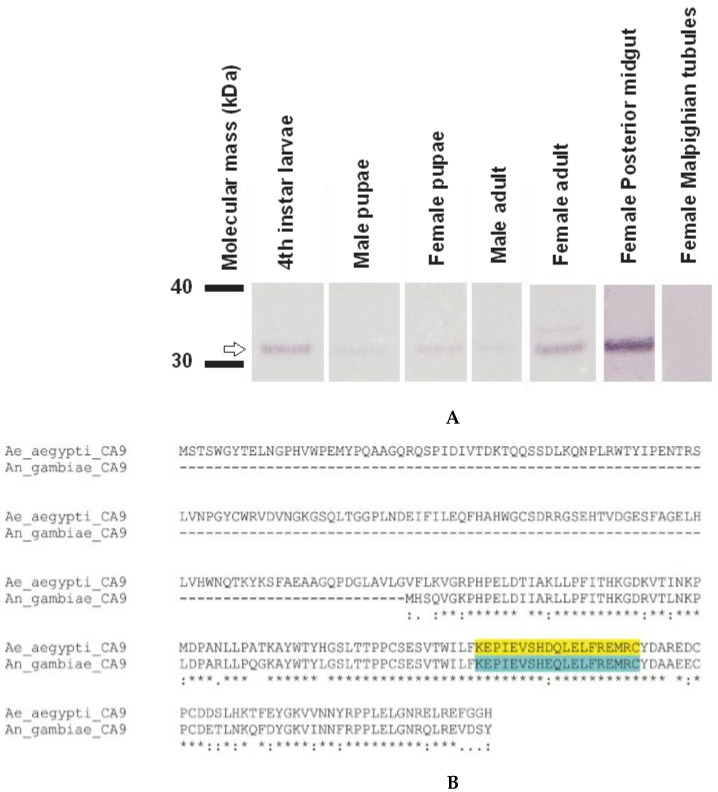
Western blot of AeCA9 in each life stage, female posterior midgut, and female Malpighian tubules. (**A**) In this western blot, the same life stages, sexes, and tissues were analyzed as in Figure 7. 6 μg of total protein was loaded for the life stages (4th instar larvae, Male pupae, Female pupae, Male adult, Female adult) and 40 μg of total protein was loaded for the female posterior midugt and female Malpighian tubules. AeCA9 was detected in all the life stage and tissue samples analyzed except the MTs (top row). There is a higher molecular weight band in the female adult sample, but that could be due to over-development; (**B**) This is an alignment showing the antigenic sequence used to develop the antisera targeting AeCA9 in (**A**) and all subsequent immunohistochemical analyses. The antisera was generated in chicken to target *Anopheles gambiae* CA9, and it has suitable cross-reactivity with *Aedes aegypti* as shown. Take note that there is only 1 amino acid difference in 19 between the antigenic sites of AgCA9 and AeCA9.

AeCA9 transcript and protein expression were investigated in each life stage, sex, the posterior midgut (PMG), and the Malpighian tubules (MTs). The MTs were included in this study because of their role in ion and water balance is linked to digestive activity in both the larvae and adults. The transcripts were analyzed via polymerase chain reaction (PCR) and the proteins via Western blots. The expression profile for AeCA9 transcripts indicates that it is expressed at each life stage and within each sex (Figure 6), giving support to the transcriptome analyses above. We also found that AeCA9 transcript was expressed in the PMG while minimal AeCA9 transcript was detected in the MTs. The number of cycles for PCR in the life stages and the PMG differed from that of the MT because it took 36 cycles to detect AeCA9 in the MT. For the western blots of AeCA9, 6 μg of total protein was loaded for the life stages and 40 μg of total protein was loaded for the PMG and MTs. AeCA9 protein was expressed in all stages/sexes analyzed (Figure 7A). Last, AeCA9 protein was present in the female PMG but was not detectable in the MTs via Western blot. An alignment was done to verify that the antigenic sequence targeted by the AgCA9 antibody aligned with the *Aedes aegypti* CA9 sequence (Figure 7B). According to this analysis, the antigenic sequence of *Aedes aegypti* is 95% identical (one amino acid difference out of 19) to *Anopheles gambiae*, which indicates that the antibody will likely target AeCA9.

### 3.5. Subcellular Compartmentalization of AeCA9 in Aedes aegypti

*Aedes aegypti* larvae and adults were sectioned and immunostained using the protocol established in Smith et al. 2007 [4]. A control using yolk from chickens before the antigens induced the production of antibodies (pre-immune control) was used in adult female tissue sections, and no background was detected. A second control was used in which only secondary antibodies (no primary antibodies) were applied to adult female tissue sections, and no signal was detected from this secondary antibody only control. Also, the AgCA9 immune IgY was blocked using the antigenic peptide in the larvae and adults, and the signal was depleted post-blocking.

#### 3.5.1. *Aedes aegypti* Larvae

**Figure 8:**
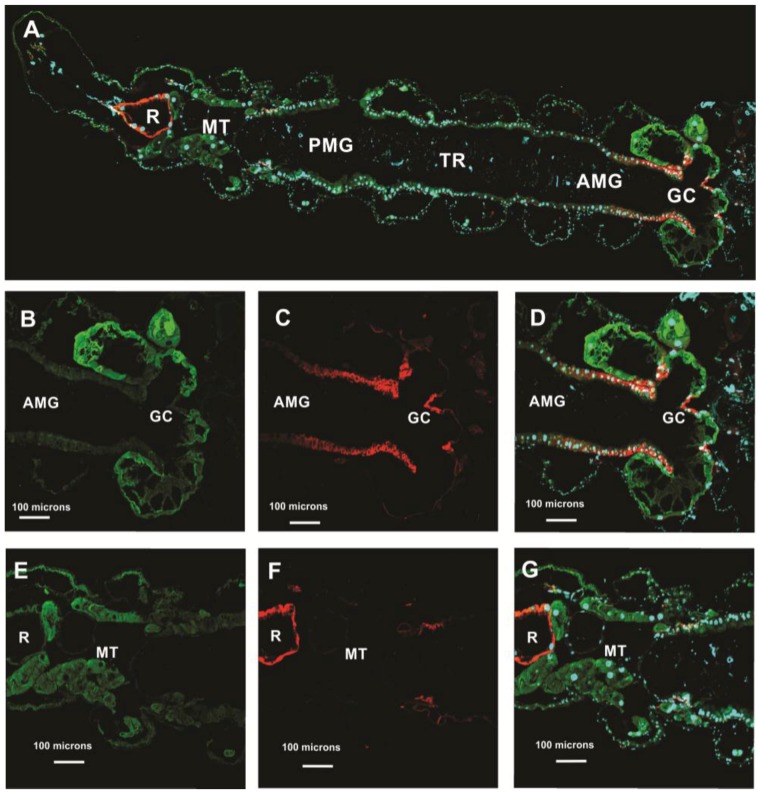
CA9 is compartmentalized to the GC and MTs of *Aedes aegypti* larvae. This figure shows transverse sections of a 4th instar larvae. The sections were immunostained with antibodies targeting the following: AeCA9 (green), NaK-ATPase (red), and nuclei were counterstained with DAPI (cyan). (**A**) A montage of the entire alimentary canal which is comprised of the following regions: Gastric caeca (GC), Anterior midgut (AMG), transitional region (TR), posterior midgut (PMG), Malpighian tubules (MTs), and rectum (R). In **B**–**D**, the GC is either immunostained with AeCA9 (**B**), NaK-ATPase (**C**), or a merge of AeCA9, NaK-ATPase, and DAPI (**D**). In **B**, AeCA9 is detected in all the cells of the GC except for the cells that make up the stem (arrowhead). AeCA9 is detected in the cap cells (arrow), and the signal for AeCA9 in the caecal lumen (*) is low. In **E**–**G**, the MTs are either immunostained with AeCA9 (**E**), NaK-ATPase (**F**), or a merge of AeCA9, NaK-ATPase, and DAPI (**G**). AeCA9 could be detected in the distal MT (arrow with tail), but not as much in the proximal MT (flagged arrow). In both parts of the MTs, signal intensity for AeCA9 is low. There was no immunoreactivity of AeCA9 in the rectum, as was previously demonstrated by Smith et al. 2008 [40]. The scale bars in A–G represent 100 microns.

In *Aedes aegypti* larvae, AeCA9 signal was detected in the midgut and MTs (Figure 8). In the GC, cap cells were distinguishable by the low signal of sodium-potassium ATPase (NaK-ATPase) [19]. Robust immunostaining for AeCA9 was detected in almost all the cells of the GC which includes the cap cells, ion transport cells, and absorptive/secretory cells. In the MTs, AeCA9 was detected in the cytoplasm of the principal cells. Other cells of the mid gut showed low levels of staining in comparison to the cells of the GC.

#### 3.5.2. *Aedes aegypti* Adults

**Figure 9:**
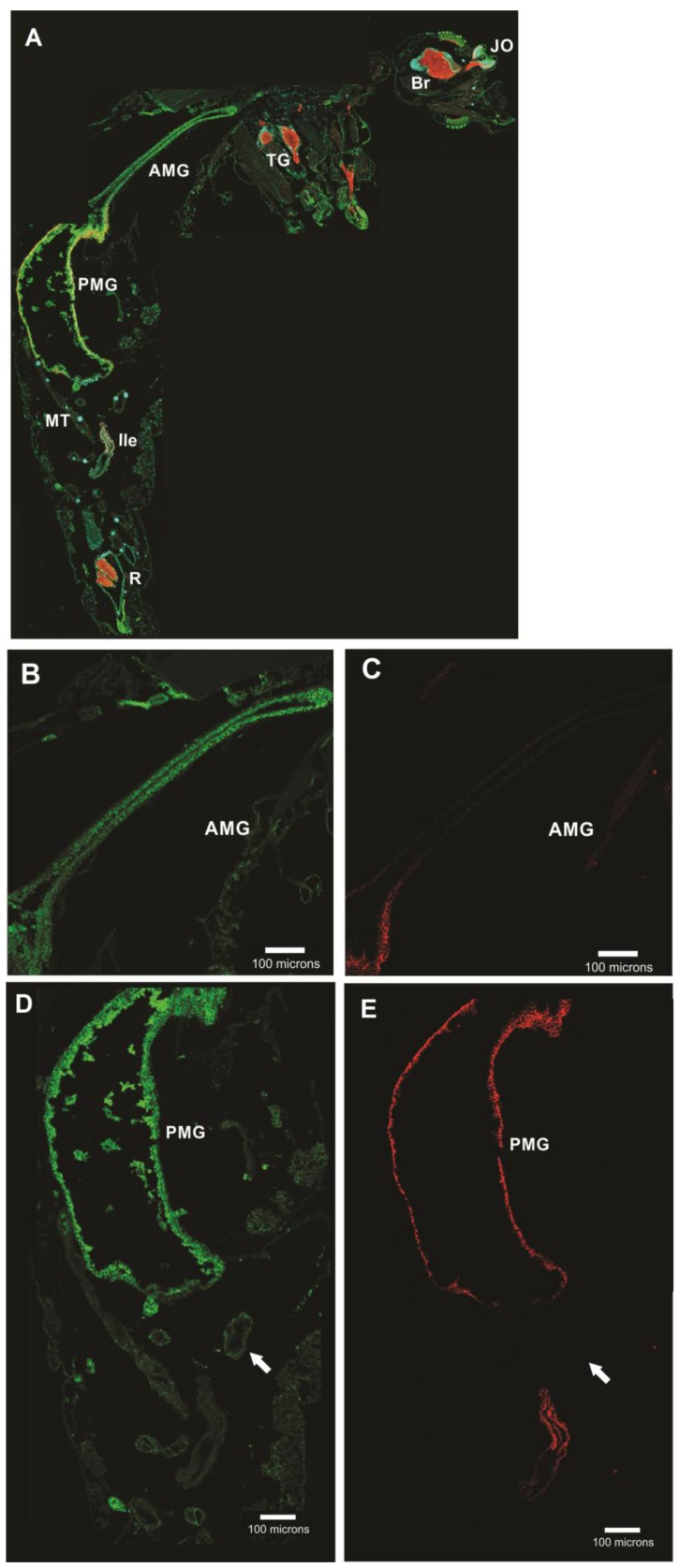
AeCA9 is compartmentalized to the female PMG and MTs. This figure shows transverse sections of a female adult. These sections were immunostained with antibodies targeting the following: AeCA9 (green), NaK-ATPase (red), and DAPI (cyan). (**A**) In the montage, the following organs can be seen: Johnston′s organ (JO), Brain (Br), thoracic ganglia (TG), anterior midgut (AMG), posterior midgut (PMG), Malpighian tubules (MT), ileum (Ile), and rectum (R); (**B**) AeCA9 is detectable in the cytoplasm throughout the length of the AMG; (**C**) NaK-ATPase is minimally expressed throughout the length of the AMG; (**D**) AeCA9 is detected in the PMG, while a faint signal for AeCA9 is detected in the MTs (arrow); (**E**) NaK-ATPase is detected on the basal membranes of the PMG epithelial cells and the ileum. The scale bars in **A**–**E** represent 100 microns.

**Figure 10:**
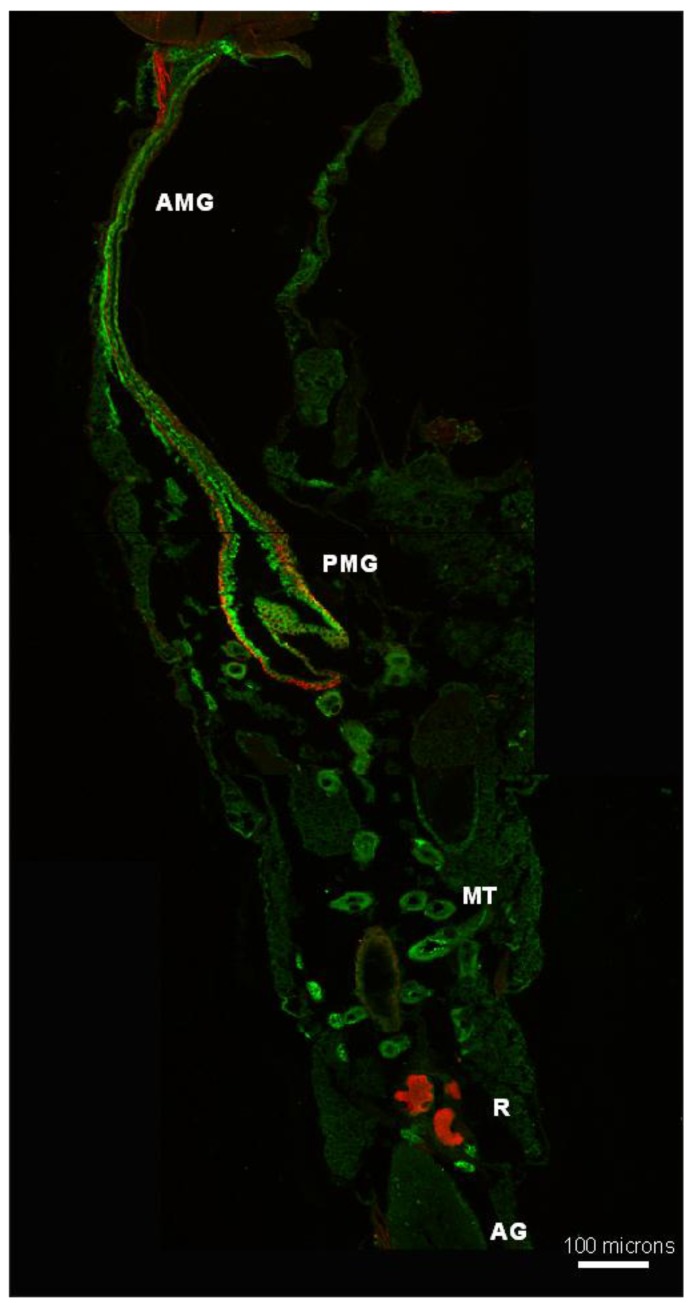
AeCA9 is compartmentalized to the alimentary canal of male *Aedes aegypti*. This image is a montage of the adult male alimentary canal, showing the anterior midgut (AMG), posterior midgut (PMG), Malpighian tubules (MT), Rectum (R), and accessory glands (AG). AeCA9 (green) and NaK-ATPase (red) are used to immunostain this section of a male mosquito, and AeCA9 signal was detected in the AMG, PMG, and MTs. NaK-ATPase was detected in the PMG, part of the AMG, and the rectal papillae.

In the adult female, AeCA9 protein signal was detected in the cytoplasm of the AMG and PMG epithelial cells (Figure 9). Also, a low signal for AeCA9 was detected in the MT principal cells. AeCA9 is localized to almost all the PMG epithelial cells, but is not evident in the cells of the pylorus. The pylorus is part of the hindgut between the PMG and ileum that connects with the MTs [20]. The hindgut regions were also analyzed, but AeCA9 was not detected in the ileum, and only a faint signal was detectable in the rectum. In the MTs, the AeCA9 signal was distributed in a punctate pattern within the cytoplasm of principal cells. Additionally, *Aedes aegypti* male mosquitoes were analyzed for the compartmentalization of AeCA9. As in the adult females, AeCA9 is readily detectable in the AMG, PMG, and the MTs (Figure 10).

**Figure 11:**
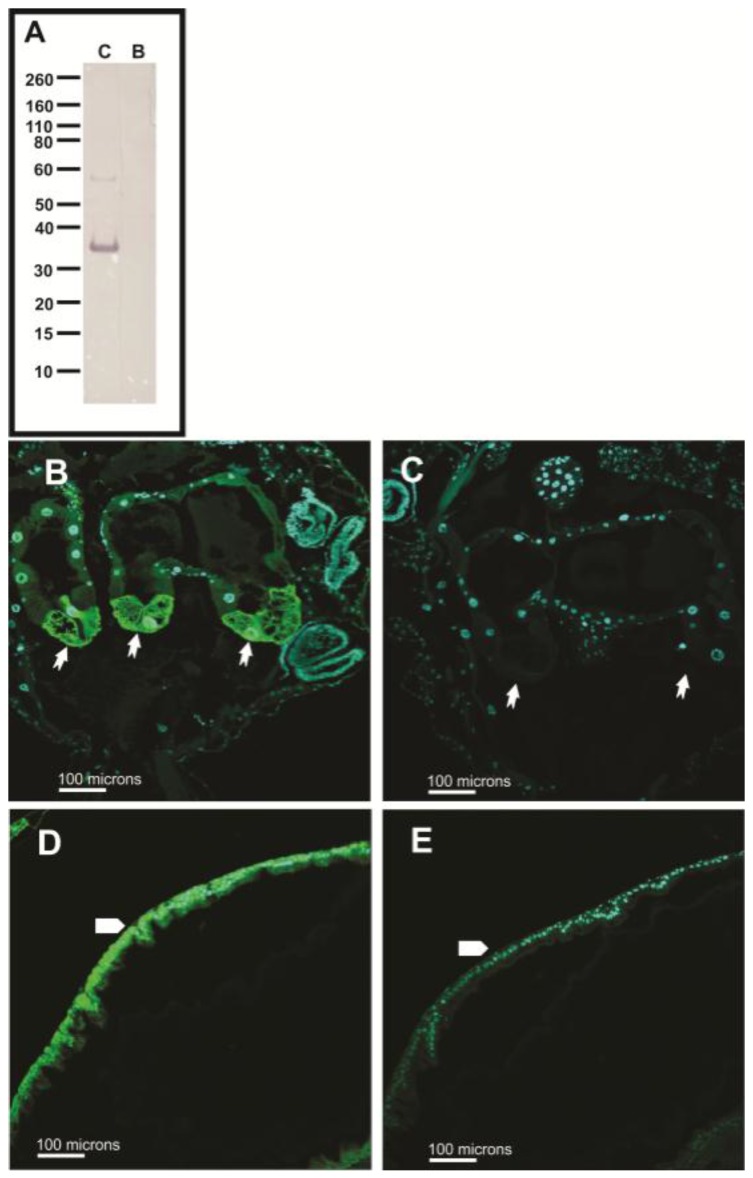
Pre-absorption controls of AeCA9 antibody targeting larvae and adults. In this figure, the AeCA9 primary antibody was pre-absorbed with its associated peptide to determine its specificity in larvae and adults. (**A**) The protein used in the western blot is isolated PMG protein. Non-absorbed antiserum targeted lane **C** (control), while absorbed antiserum targeted lane **B** (blocked/absorbed). AeCA9 is around 32 kDa. AeCA9 was detected using the control antiserum, and the blocked antiserum did not detect AeCA9; (**B**–**E**) In the sections, either larval GC (**B** & **C**) or the adult PMG (**D** & **E**) were targeted. The same antibody solution sets were used in the control (**B** & **D**) and blocked (**C** & **E**) tissue sections and western blots. In both the larval GC and adult PMG, AeCA9 signal was blocked after preabsorption with the peptide. In the larval GC, arrows indicate cap cells. In the adult PMG, the arrows represent the epithelial cells of the PMG. The green signal is for AeCA9 while the cyan signal is for DAPI.

Pre-absorption controls were used to determine the specificity of the AgCA9 IgY antibody (Figure 11). This combination of antibody and peptide successfully blocked antibody binding to *Anopheles gambiae* larvae [4]. In *Aedes aegypti*, the signal was successfully blocked as measured by Western blot (Figure 11A) and Immunohistochemical analysis (Figure 11B–E). The higher molecular weight bands from the Western blot (lane C in Figure 11A) were not detected post-absorption, which indicates these higher molecular weight bands were probably aggregates containing AeCA9. To verify the antibody does not bind to non-specific targets, the antigenic sequence was blasted against the entire *Aedes aegypti* genome. Only two proteins other than CA showed any alignment with the target peptide by six to seven amino acids, but the e-values were very high (>0.1) which indicates the matches detected on the alignment are most likely due to random chance.

## 4. Discussion

In this study, we analyzed the genomic, transcript, and protein sequences of each CA isoform in *Aedes aegypti*. We predicted the subcellular compartmentalization of all 14 CA sequences using motif analysis software. Predictively, five CAs are cytoplasmic, four CAs have signal peptides, three CAs are compartmentalized to the membrane, and two CAs are mitochondrial. Next, the phylogeny generated against the 14 CA proteins suggest AeCA9 most closely resembles the common ancestral CA that evolved before the protostome-deuterostome split [4]. We also analyzed the developmental transcriptome of *Aedes aegypti* [6] and determined that *AeCA9*, *AeCA7* (both variants), *AAEL009330*, *AeCA3* (both variants), *AeCAβ*, *AeCA6*, *AeCA10*, and *AeCA4* were abundantly expressed in the different life stages and post blood meal compared with the remaining sic CA isoforms. AeCA9, a focus of this manuscript, expressed mRNA at all developmental stages, sexes, and within the PMG of adult females. We also analyzed the protein compartmentalization of AeCA9 using Western blots and confocal microscopy. Our analysis shows that AeCA9 protein is expressed in the larval midgut and MT of the alimentary canal. The GC cells exhibit particularly robust immunolocalization of AeCA9. In adult females and males, AeCA9 is expressed in the AMG, PMG, and MT. This study also showed that isoforms of CA that resemble the most ancestral mosquito CAs (AeCA9 and AeCAβ) are the most widely expressed CAs both spatially and temporally. In addition, the adult life stage and larvae express AeCA9 in digestive and ion regulatory tissues, suggesting a similarity in function for this isoform across two dynamic life stages.

### 4.1. AeCA9 Resembles the Most Ancestral Alpha CA and Is Widely Expressed in Aedes aegypti

From the molecular phylogeny generated in Figure 5, AeCA9 clusters with most of the Human CAs and its orthologs in each of the other species analyzed. The remaining alpha CAs from *Drosophila*, Culicine mosquitoes, and Anopheline mosquitoes did not cluster with AeCA9. This pattern of insect CA segregation from AeCA9 was observed in the Smith et al. 2007 phylogeny [4]. The updated phylogeny in this study supports the inference from Smith et al. [4] that AeCA9 is most similar to the isoform of CA from which the insect-specific alpha CAs derived.

AeCA9 is also the most widely expressed isoform in all the life stages and is abundantly expressed post-blood meal (Figure 2 and Figure 3), yet there is a rich diversity of insect-specific CA proteins in the genome of *Aedes aegypti*. According to the Akbari transcriptome, insect-specific CAs are expressed within restricted developmental windows, physiological processes, or tissue compartments (Figure 2, Figure 3 and Figure 4). The spatially and temporally restricted expression of insect-specific CAs supports the hypothesis that insect CAs duplicated to take on diverse functions as specialized compartments evolved. Similar instances of CA specialization and gene duplication have been recorded in other more basal taxa [45]. AeCA9 is intertwined with a multitude of physiological processes, but the midgut is the region of focus of this manuscript.

### 4.2. AeCA9 Is Compartmentalized to the Alimentary Canal in Both Larvae and Adults

In this study, we determined that AeCA9 protein is expressed in the alimentary canal of larvae and adults. The specific importance of gut AeCA9 remains unclear in *Aedes aegypti*, but the enzymatic products of CA activity (CO_2_, HCO_3_^−^ and H^+^ ) play roles in pH regulation, ion transport, fluid secretion, and amino acid symport as well as the obvious elimination of metabolic waste products (i.e., CO_2_). Although inhibition of CA activity in the intact or semi intact animal can produce acidification of the alkaline midgut [15,16] isolated and perfused AMG and/or PMG do not require local CA activity to alkalinize the gut lumen [18,27]. Pharmacological studies show that the primary motive force of pH alkalinization in the AMG is by the action of the H^+^V-ATPase (ibid) which is apically localized in AMG epithelial cells [28] and thus “pulling” protons out of the AMG lumen. CO_2_/HCO_3_^−^/CO_3_^2−^ and hence CA may play a passive role in providing the major buffering ions relative to hi or low H^+^ concentrations in the different regions of the gut lumen. The apparent accumulation of CA protein in GC cells, which is distinctly higher than other gut cells, suggests that the GC are a source of HCO_3_^−^/CO_3_^2−^ which may either move into the gut lumen for eventual excretion or into the hemolymph for respiratory elimination. If specific bicarbonate transporters are positioned such that a net movement of HCO_3_^−^/CO_3_^2−^ is into the caecal lumen and thus to the AMG lumen, then lumenal pH will balance relative to the final concentration of both H^+^ and HCO_3_^−^/CO_3_^2−^. Recent analyses of certain members of the SLC4 bicarbonate transporter protein family do in fact show that such bicarbonate transporters are expressed in larval mosquito caeca epithelial cells [19]. The elegant physiological analyses of isolated and perfused midgut (minus the GC) clearly show that H^+^V-ATPase can drive alkalinization in the absence of CA activity [18,27] but other studies on living intact and semi intact preparations show that more processes, including the overall disposition of the products of CA activity, influence pH regulation in the whole animal [5].

In the MTs of larvae, AeCA9 was detected in the principal cells and not the stellate cells, similar to that of *Anopheles gambiae* larvae [4] (Figure 8). AeCA9 was also detected in the principal cells of female and male adult MTs (Figure 9 and Figure 10). More specifically, this work shows that AeCA9 is compartmentalized to intracellular vesicles in the principal cells of adult females. AeCA9 is also likely found in the cytoplasm of female adult MTs as the Piermarini model suggests [33]. The Piermarini model predicts metabolic CO_2_ is rapidly converted to HCO_3_^−^ and H^+^ via a cytoplasmic CA and that the H^+^ are utilized by H^+^V-ATPase on the apical microvilli of MT principal cells (ibid). The model also suggests that HCO_3_^−^ moves into the stellate cells via gap junctions to be transported out into the hemolymph via the basally localized AE1 (ibid). AeCA9 possibly provides some of the H^+^ for H^+^ V-ATPases and HCO_3_^−^ for stellate cell AE1 as the Piermarini model suggests [19,33]. CAs could also play a role in the transport of ions in CaCO_3_^-^ containing storage vesicles of MT principal cells. CA activity has been detected in MT transport vesicles in *Drosophila melanogaster* and *Culex quinquefasciatus* [46,47]. In the Wessing et al. 1997 study [46], CA activity was also inhibited using sulfonamides, and this resulted in a reduction in ion transport processes. The exact function of AeCA9 in Aedes malpighian tubules is difficult to determine without knockdown assays, but supporting evidence from previous studies suggests AeCA9 plays a role in storage vesicle function and chloride transport.

AeCA9 was also detected in the epithelial cells of the PMG in female adults (Figure 9). We hypothesize that the products of AeCA9 activity play roles in pH regulation, fluid secretion, and amino acid transport [23,48]. This hypothesis is adapted from the model proposed by Pacey and O′Donnell [32]. AeCA9 may provide H^+^ for the transepithlial potential established by H^+^ V-ATPase, which drives the displacement of ions across biological membranes for pH and ion regulatory processes. This transepithelial potential may also be utilized by putative H^+^/amino acid symporters for the intake of amino acids post-blood meal [48]. Finally, Onken and Moffett determined that CA plays a role in fluid absorption reflected in the 60%–70% reduction in fluid absorption after inhibiting CA activity using acetazolamide [17]. All of these models propose the involvement of a CA, yet none propose which isoform of CA plays a role due to a lack of knowledge of CA isoform expression and compartmentalization. With AeCA9 localized to the PMG epithelial cells, this study is the first to propose a gene for CA in the processes of pH regulation, fluid secretion, and amino acid transport in *Aedes aegypti*. AeCA9 was also localized to the male gut, but its role is unclear as functional studies have not been conducted on the role of AeCAs in the gut of adult males. Hypothetically, AeCAs could be playing a role in CO_2_ regulatory processes in the male gut epithelial cells.

The localization of AeCA9 to intracellular vesicles within the principle cells of female adult MTs was not consistent across all methodologies. The transcript levels for AeCA9 in the MTs were minimal, while no protein could be detected in the female MTs using western blots. Yet, Immunohistochemical analyses indicated AeCA9 was expressed in the transport vesicles of the MTs in adult females along with larvae and male adults. There are a couple factors that could explain this discrepancy. First, the expression patterns for AeCA9 could be wildly different between life stages (larvae vs. female adults) and sex (male vs. female adults) due to the different habitats and diets associated with each life stage. Another factor causing a discrepancy in expression is the variability in sensitivity of each method used. Western blots and Immunohistochemical analyses of tissues may give different results due to factors such as solubility of proteins in western blots, sensitivity of enzyme-based detection methods (alkaline phosphatase), and quantity of proteins analyzed. Immunohistochemical analyses of tissues may be more sensitive because the antibody is binding directly to the protein in the tissues with no need for extraction.

## 5. Conclusions

AeCA9 resembles the most closely related alpha CA to the presumed common ancestral CA between Anophelines and Culicines, and is likely similar to the CA from which the insect-specific CAs evolved. We used molecular phylogenetics, data mining of transcriptomes, molecular biology, and immunohistochemistry to characterize AeCA9 as a soluble cytoplasmic protein in the adult and larval alimentary canal. This work gives greater resolution to the mechanisms of pH regulation and proton transport from past studies [3,32]. Del Pilar Corena et al. [3] suggested that CAs play a role in pH regulation of the gut, and these current studies show that AeCA9 is one of the CAs found in the gut tissues. Pacey et al. [32] showed that H^+^ transport post-blood meal could be inhibited in the gut with acetazolamide, and our results suggest that AeCA9 is likely one of the CAs inhibited by acetazolamide in the Pacey et al. study [32]. This implies AeCA9 is involved in regulating H^+^ transport in the PMG post-blood meal. Finally, methazolamide inhibited fluid absorption in the isolated adult PMG, which demonstrates a role for CAs in osmoregulatory processes during blood meal processing [17]. A caveat to this study is that more than one CA is expressed inside the gut, and they are also most likely involved with pH regulatory and ion transport processes. Elucidating their functions is an important step to understanding the molecular underpinnings of pH metabolic CO_2_ regulation in the alimentary canal of *Aedes aegypti*. Deeper understanding of the molecular mechanisms that regulate alimentary canal function and general physiological homeostasis provides expanded targeting strategies for controlling mosquito populations into the future.

## Figures and Tables

**Table 1:** Carbonic anhydrase genes in *Aedes aegypti*.

Isoform	Genome Sequence Length	Transcript Sequence Length	5′ UTR Length	3′ UTR Length	Coding Sequence Length	Amino Acids
AAEL000617	6274	1452	0	0	1452	483
**AAEL000816**	**1015**	**948**	**36**	**261**	**651**	**216**
AAEL000843	877	822	0	0	822	273
AAEL003644	986	986	0	0	987	328
**AAEL004930**	**54,321**	**2742**	**801**	**1110**	**831**	**276**
AAEL005337	32,439	1530	0	486	1044	347
AAEL005520-RA	16,994	1082	147	164	771	256
AAEL005520-RB	16,994	1567	632	164	771	256
AAEL008031	68,384	999	0	0	999	332
AAEL008828	34,026	1737	444	348	945	314
AAEL009323	28,262	1276	0	382	894	297
AAEL009330	1157	1103	102	53	948	315
AAEL010886	17,765	1657	509	125	1023	340
AAEL010893-RA	34,039	1773	253	380	1140	379
AAEL010893-RB	34,039	2215	695	380	1140	379
AAEL010894	4503	1522	562	105	855	284

All nucleotide sequence lengths are in base pairs, while protein sequence lengths are shown in amino acids. The sequence AAEL000816 in bold is a beta CA, a distinct family of carbonic anhydrases from the alpha CAs. The sequence AAEL004930 is in bold and blue as it is the focus of this manuscript.

**Table 2:** Carbonic anhydrase proteins in *Aedes aegypti*.

Isoform	Signal Peptide	Transmembrane Helix	Predicted Localization	Predicted Orthology	*Aegypti* Identifiers
AAEL000617	No	No	Cytoplasmic	AgCA-RP5	AeCA-RP5
**AAEL000816**	**No**	**No**	**Cytoplasmic**	**AgCAβ**	**AeCAβ**
AAEL000843	No	No	Cytoplasmic	AGAP013402	AAEL000843
AAEL003644	No	No	Cytoplasmic	AgCA8	AeCA8
**AAEL004930**	**No**	**No**	**Cytoplasmic**	**AgCA9**	**AeCA9**
AAEL005337	No	Yes	Integral membrane	AgCA4	AeCA4
AAEL005520	No	No	Mitochondrial	AgCA3/11	AeCA3/11
AAEL008031	No	No	Cytoplasmic	AgCA-RP2	AeCA-RP2
AAEL008828	Yes	No	Secreted	AgCA6	AeCA6
AAEL009323	Yes	Yes	GPI-anchored	AgCA10	AeCA10
AAEL009330	Yes	Yes	GPI-anchored	N/A	AAEL009330
AAEL010886	Yes	No	Secreted	AGAP002360	AAEL010886
AAEL010893	Yes	No	Secreted	AgCA7	AeCA7
AAEL010894	Yes	No	Secreted	AGAP013402	AAEL010894

The Predicted orthology (Ortho) of each isoform to *Anopheles gambiae* is listed. The *Anopheles gambiae* ortholog identifiers (AgCAX) are based on identifiers established by [4]. Orthologous sequences without an AgCA identifier were labeled with the full Vectorbase identifier AGAP0XXXXX. The Aegypti identifiers (AeCAX) are based on identifiers established by [4]. *Aedes aegypti* sequences without a corresponding AgCA identifier were labeled with the full Vectorbase identifier AAEL0XXXXX. The sequence AAEL000816 in bold is a beta CA, a distinct family of carbonic anhydrases from the alpha CAs. The sequence AAEL004930 is in bold and blue as it is the focus of this manuscript.

## Supplementary Materials

The following are available online at www.mdpi.com/1660-4601/14/2/213/s1, Figure S1: Alignment of AeCA3 splice variants, Figure S2: Alignmnet of AeCA7 splice variants.

## References

[1] Noriega F.G., Edgar K.A., Bechet R., Wells M.A. (2002). Midgut exopeptidase activities in *Aedes aegypti* are induced by blood feeding. J. Insect Physiol..

[2] Dadd R.H. (1975). Alkalinity within the midgut of mosquito larvae with alkaline-active digestive enzymes. J. Insect Physiol..

[3] Del Pilar Corena M., VanEkeris L., Salazar M.I., Bowers D., Fiedler M.M., Silverman D., Tu C., Linser P.J. (2005). Carbonic anhydrase in the adult mosquito midgut. J. Exp. Biol..

[4] Smith K.E., VanEkeris L.A., Linser P.J. (2007). Cloning and characterization of AgCA9, a novel alpha-carbonic anhydrase from *Anopheles gambiae* Giles sensu stricto (Diptera: Culicidae) larvae. J. Exp. Biol..

[5] Linser P.J., Smith K.E., Seron T.J., Neira Oviedo M. (2009). Carbonic anhydrases and anion transport in mosquito midgut pH regulation. J. Exp. Biol..

[6] Akbari O.S., Antoshechkin I., Amrhein H., Williams B., Diloreto R., Sandler J., Hay B.A. (2013). The developmental transcriptome of the mosquito *Aedes aegypti*, an invasive species and major arbovirus vector. G3.

[7] McKenna R., Frost S.C. (2014). Overview of the carbonic anhydrase family. Subcell. Biochem..

[8] De Simone G., Di Fiore A., Capasso C., Supuran C.T. (2015). The zinc coordination pattern in the η-carbonic anhydrase from *Plasmodium falciparum* is different from all other carbonic anhydrase genetic families. Bioorg. Med. Chem. Lett..

[9] Hilvo M., Baranauskiene L., Salzano A.M., Scaloni A., Matulis D., Innocenti A., Scozzafava A., Monti S.M., Di Fiore A., De Simone G. (2008). Biochemical characterization of CA IX, one of the most active carbonic anhydrase isozymes. J. Biol. Chem..

[10] Lindskog S. (1997). Structure and mechanism of carbonic anhydrase. Pharmacol. Ther..

[11] Buren S., Ortega-Villasante C., Blanco-Rivero A., Martinez-Bernardini A., Shutova T., Shevela D., Messinger J., Bako L., Villarejo A., Samuelsson G. (2011). Importance of post-translational modifications for functionality of a chloroplast-localized carbonic anhydrase (CAH1) in Arabidopsis thaliana. PLoS ONE.

[12] Purkerson J.M., Schwartz G.J. (2007). The role of carbonic anhydrases in renal physiology. Kidney Int..

[13] Postel R., Sonnenberg A. (2012). Carbonic anhydrase 5 regulates acid-base homeostasis in zebrafish. PLoS ONE.

[14] Henry R.P., Swenson E.R. (2000). The distribution and physiological significance of carbonic anhydrase in vertebrate gas exchange organs. Resp. Physiol..

[15] Del Pilar Corena M., Fiedler M.M., Van Ekeris L., Tu C., Silverman D.N., Linser P.J. (2004). Alkalization of larval mosquito midgut and the role of carbonic anhydrase in different species of mosquitoes. Comp. Biochem. Physiol. C Toxicol. Pharmacol..

[16] Boudko D.Y., Moroz L.L., Harvey W.R., Linser P.J. (2001). Alkalinization by chloride/bicarbonate pathway in larval mosquito midgut. Proc. Natl. Acad. Sci. USA.

[17] Onken H., Moffett D.F. (2015). Fluid absorption in the isolated midgut of adult female yellow fever mosquitoes (*Aedes aegypti*). J. Exp. Biol..

[18] Onken H., Moffett S.B., Moffett D.F. (2008). Alkalinization in the isolated and perfused anterior midgut of the larval mosquito *Aedes aegypti*. J. Insect Sci..

[19] Linser P.J., Neira Oviedo M., Hirata T., Seron T.J., Smith K.E., Piermarini P.M., Romero M.F. (2012). Slc4-like anion transporters of the larval mosquito alimentary canal. J. Insect Physiol..

[20] Clements A.N. (1992). The Biology of Mosquitoes.

[21] Volkmann A., Peters W. (1989). Investigations on the midgut caeca of mosquito larvae-II. Functional aspects. Tissue Cell.

[22] Moffett S.B., Moffett D.F. (2005). Comparison of immunoreactivity to serotonin, FMRFamide and SCPb in the gut and visceral nervous system of larvae, pupae and adults of the yellow fever mosquito *Aedes aegypti*. J. Insect Sci..

[23] Patrick M.L., Aimanova K., Sanders H.R., Gill S.S. (2006). P-type Na+/K+-ATPase and V-type H+-ATPase expression patterns in the osmoregulatory organs of larval and adult mosquito *Aedes aegypti*. J. Exp. Biol..

[24] Seron T.J., Hill J., Linser P.J. (2004). A GPI-linked carbonic anhydrase expressed in the larval mosquito midgut. J. Exp. Biol..

[25] Cocke J., Bridges A.C., Mayer R.T., Olson J.K. (1979). Morphological effects of insect growth regulating compounds on *Aedes aegypti* (Diptera: Culicidae) larvae. Life Sci..

[26] Corena M., Seron T.J., Lehman H.K., Ochrietor J.D., Kohn A., Tu C., Linser P.J. (2002). Carbonic anhydrase in the midgut of larval *Aedes aegypti*: Cloning, localization and inhibition. J. Exp. Biol..

[27] Jagadeshwaran U., Onken H., Hardy M., Moffett S.B., Moffett D.F. (2010). Cellular mechanisms of acid secretion in the posterior midgut of the larval mosquito (*Aedes aegypti*). J. Exp. Biol..

[28] Zhuang Z., Linser P.J., Harvey W.R. (1999). Antibody to H^+^V-ATPase subunit E colocalizes with portasomes in alkaline larval midgut of freshwater mosquito (*Aedes aegypti*). J. Exp. Biol..

[29] Foster W.A. (1995). Mosquito sugar feeding and reproductive energetics. Annu. Rev. Entomol..

[30] Okuda K., Caroci A., Ribolla P., Marinotti O., de Bianchi A.G., Bijovsky A.T. (2005). Morphological and enzymatic analysis of the midgut of Anopheles darlingi during blood digestion. J. Insect Physiol..

[31] Pullikuth A.K., Aimanova K., Kangethe W., Sanders H.R., Gill S.S. (2006). Molecular characterization of sodium/proton exchanger 3 (NHE3) from the yellow fever vector, *Aedes aegypti*. J. Exp. Biol..

[32] Pacey E.K., O′Donnell M.J. (2014). Transport of H(+), Na(+) and K(+) across the posterior midgut of blood-fed mosquitoes (*Aedes aegypti*). J. Insect Physiol..

[33] Piermarini P.M., Grogan L.F., Lau K., Wang L., Beyenbach K.W. (2010). A SLC4-like anion exchanger from renal tubules of the mosquito (*Aedes aegypti*): Evidence for a novel role of stellate cells in diuretic fluid secretion. Am. J. Physiol. Regul. Integr. Comp. Physiol..

[34] Giraldo-Calderón G.I., Emrich S.J., MacCallum R.M., Maslen G., Dialynas E., Topalis P., Ho N., Gesing S., Consortium T.V., Madey G. (2015). VectorBase: An updated bioinformatics resource for invertebrate vectors and other organisms related with human diseases. Nucleic Acids Res..

[35] Mitchell A., Chang H.Y., Daugherty L., Fraser M., Hunter S., Lopez R., McAnulla C., McMenamin C., Nuka G., Pesseat S. (2015). The InterPro protein families database: The classification resource after 15 years. Nucleic Acids Res..

[36] Kall L., Krogh A., Sonnhammer E.L. (2004). A combined transmembrane topology and signal peptide prediction method. J. Mol. Biol..

[37] Horton P., Park K.J., Obayashi T., Fujita N., Harada H., Adams-Collier C.J., Nakai K. (2007). WoLF PSORT: Protein localization predictor. Nucleic Acids Res..

[38] Tamura K., Stecher G., Peterson D., Filipski A., Kumar S. (2013). MEGA6: Molecular evolutionary genetics analysis version 6.0. Mol. Biol. Evol..

[39] Edgar R.C. (2004). MUSCLE: Multiple sequence alignment with high accuracy and high throughput. Nucleic Acids Res..

[40] Smith K.E., VanEkeris L.A., Okech B.A., Harvey W.R., Linser P.J. (2008). Larval anopheline mosquito recta exhibit a dramatic change in localization patterns of ion transport proteins in response to shifting salinity: A comparison between anopheline and culicine larvae. J. Exp. Biol..

[41] Lebovitz R.M., Takeyasu K., Fambrough D.M. (1989). Molecular characterization and expression of the (Na+ + K+)-ATPase alpha-subunit in *Drosophila melanogaster*. EMBO J..

[42] Juneja P., Osei-Poku J., Ho Y.S., Ariani C.V., Palmer W.J., Pain A., Jiggins F.M. (2014). Assembly of the genome of the disease vector *Aedes aegypti* onto a Genetic linkage map allows mapping of genes affecting disease transmission. PLoS Negl. Trop. Dis..

[43] Eisenhaber B., Bork P., Eisenhaber F. (1999). Prediction of potential GPI-modification sites in proprotein sequences. J. Mol. Biol..

[44] Fisher S.Z., Tariku I., Case N.M., Tu C., Seron T., Silverman D.N., Linser P.J., McKenna R. (2006). Expression, purification, kinetic, and structural characterization of an alpha-class carbonic anhydrase from *Aedes aegypti* (AaCA1). Biocim. Biophys. Acta.

[45] Le Roy N., Jackson D., Marie B., Ramos-Silva P., Marin F. (2014). The evolution of metazoan α-carbonic anhydrases and their roles in calcium carbonate biomineralization. Front. Zool..

[46] Wessing A., Zierold K., Bertram G. (1997). Carbonic anhydrase supports electrolyte transport in Drosophila Malpighian tubules. Evidence by X-ray microanalysis of cryosections. J. Insect Physiol..

[47] Palatroni P., Gabrielli M.G., Scattolini B. (1981). Histochemical localization of carbonic anhydrase in Malpighian tubules of *Culex pipiens*. Experientia.

[48] Evans A.M., Aimanova K.G., Gill S.S. (2009). Characterization of a blood-meal-responsive proton-dependent amino acid transporter in the disease vector, *Aedes aegypti*. J. Exp. Biol..

