# Supplementary material for: Characterization of Carbonic Anhydrase 9 in the Alimentary Canal of Aedes aegypti and Its Relationship to Homologous Mosquito Carbonic Anhydrases
Source: Int J Environ Res Public Health. 2017 Feb 21;14(2):213. doi: 10.3390/ijerph14020213 (PMC5334767; doi:10.3390/ijerph14020213)
Supplement: Supplementary file 1 [file ijerph-14-00213-s001.pdf]

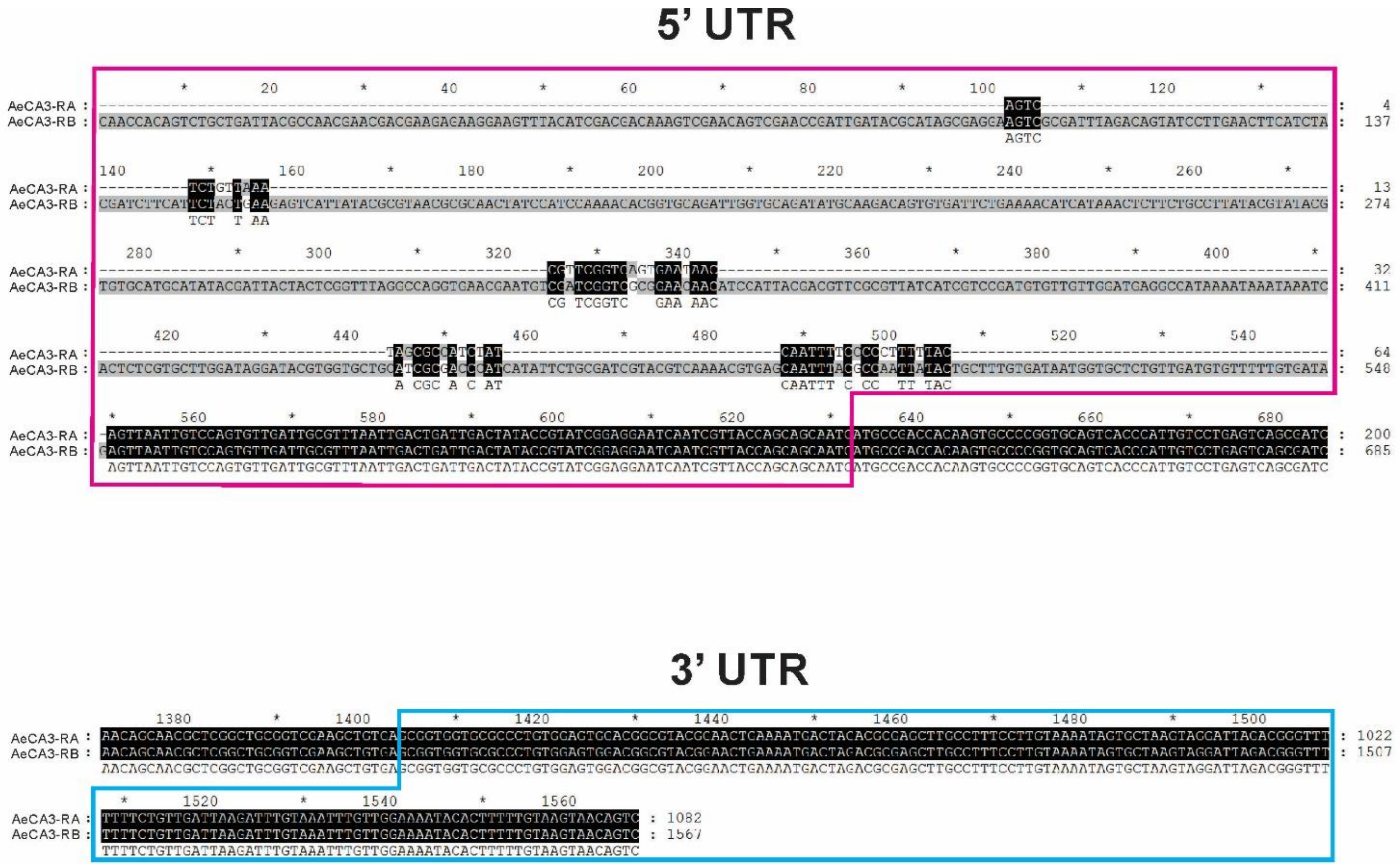

Figure S1. Alignment of AeCA3 splice variants. This figure shows only the alignments of the 5′ and 3′ UTR. The nucleotide sequences highlighted in black are identical while nucleotide sequences highlighted in grey are similar. The 5′ UTR is indicated by a magenta box, while the 3′ UTR is indicated by a light blue boc. The 5′ UTRs between both variants are divergent except for the last 83 bp.

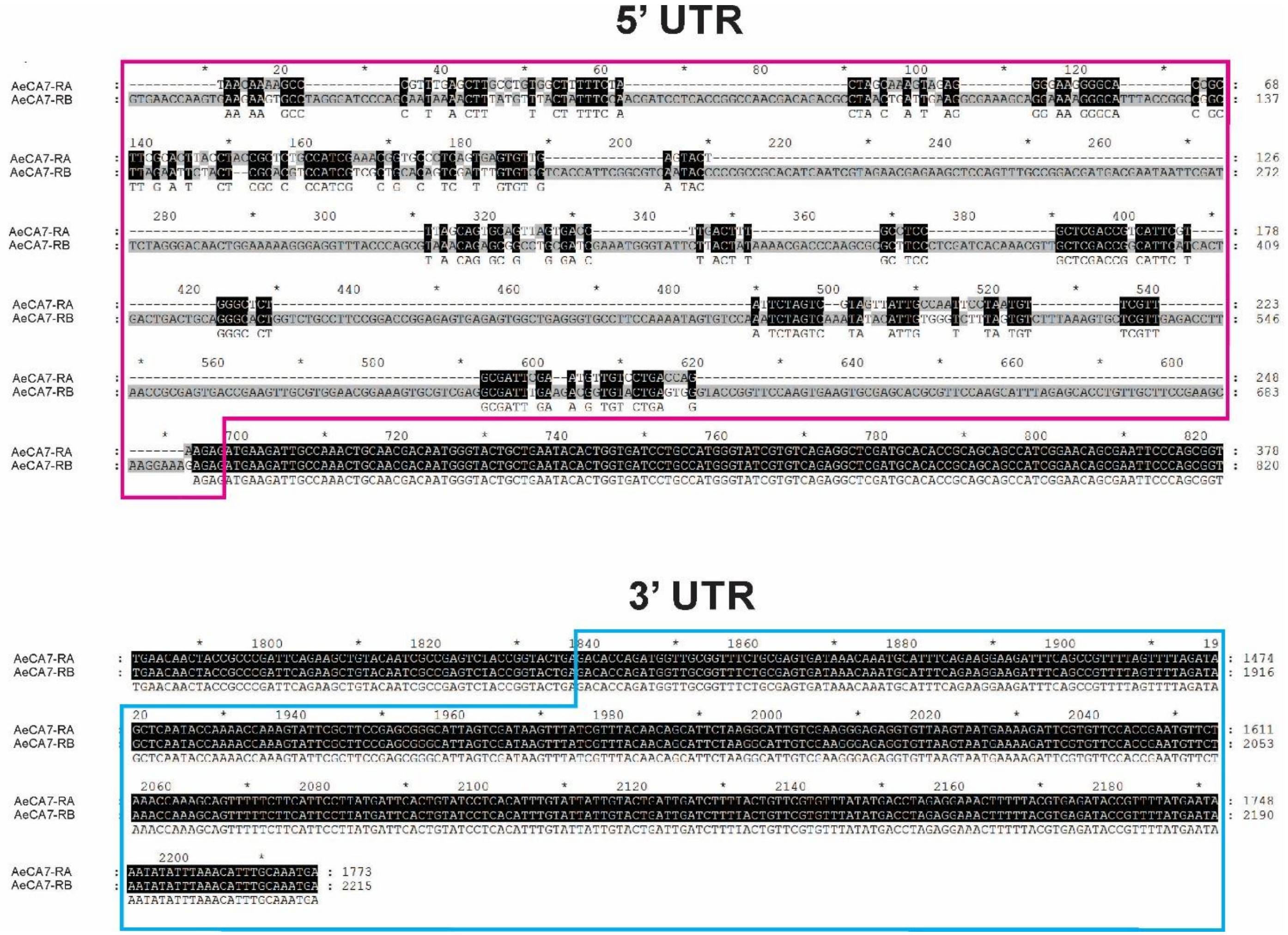

Figure S2. Alignment of AeCA7 splice variants. This figure shows only the alignments of the 5′ and 3′ UTR. The nucleotide sequences highlighted in black are identical while nucleotide sequences highlighted in grey are similar. The 5′ UTR is indicated by a magenta box while the 3′ UTR is indicated by a light blue box. The 5′ UTRs between both sequences are not similar to each other.
